# Supplementary material for: A micro-dispenser for long-term storage and controlled release of liquids
Source: Nat Commun. 2019 Jan 14;10:189. doi: 10.1038/s41467-018-08091-z (PMC6331589; doi:10.1038/s41467-018-08091-z)
Supplement: Supplementary file 1 — Supplementary information [file 41467_2018_8091_MOESM1_ESM.pdf]

Supplementary Information

A micro-dispenser for long-term storage and controlled release of liquids

Amin Kazemzadeh [a], Anders Eriksson [b], Marc Madou [c, d], Aman Russom [a]

[a] Division of Nanobiotechnology, Department of Protein Sciences, Science for Life Laboratory, KTH Royal Institute of Technology, Stockholm, Sweden.
[b] School of Engineering Sciences, Mechanics, KTH Royal Institute of Technology, Stockholm, Sweden
[c] Department of Mechanical and Aerospace Engineering, University of California, 92697 Irvine, United States.
[d] Department of Mechanical Engineering, University of California, 92697 Irvine, United States.

Supplementary Note 1:

Our results include human-based error in measuring the volume of liquid released at a given pressure as we use image-based analysis to measure the amount of liquid released from the container. For this purpose, we import our photos in ImageJ and Photoshop software and observe the liquid movement in the micropipette. Therefore, the accuracy reported for aliquoting is considered as preliminary results that need to be calibrated using sophisticated measurement methods. At this stage we intend to show the ability of consistent and sequential release of micro and nano liter of liquid. In order to evaluate the aliquoting performance we experimentally investigated the dispensing accuracy of 4 identical micro-dispensers. The length, the inner and outer diameter of the micro-dispenser container is ~ 29.5, 1 and 1.9 mm, respectively. The inner and the outer diameter of the membrane is 0.79 and 2.38 mm, respectively. In order to approximately measure the volume of liquid released we used ImageJ and Photoshop to measure the reduction of liquid along the micro-pipette. For example, we used each sub-figure shown in Supplementary Figure 1 and uses image processing to measure the liquid displacement.

Supplementary Note 2: Hemocytometry test

The absorbance of red blood cells in the plasma sample using 575 nm wavelength shows that the blood plasma collected by IDA has 10 % lower deviation from the blank control. Note that for the conventional centrifugation method we used Eppendorf Centrifuge 5810 R machine and spun the sample at 900xg for 10 minutes. We used the same setting for blood plasma separation using IDA but we increased the speed to 1400xg for approximately 2 minutes to stretch the membrane and dispense the plasma into a separate tube. Supplementary Figure 2 shows the images from plasma samples collected using micro-dispenser after carrying out hemocytometry test. We perform the experiment three times and aspired 3x15 µl of plasma for each experiment. The hemocytometry test shows no cells in the plasma sample.

Supplementary Note 3: Sequential release of liquids

In Supplementary Figure 3a we show a schematic of the whole system used in the experiment. Supplementary Figure 3b shows images of a rotating lab-on-disc that incorporates a micro-dispenser filled with red-dyed di-water. The micro-dispenser is connected via microchannels to separate receiving chambers. One of the chambers is filled with blue-dyed di-water resembling lyophilized materials in order to enhance the visualization of adding water. The micro-dispenser used in this experiment has two apertures covered with two membranes as seen in the inset figure. The membranes are pre-stressed such that they do not ever stretch simultaneously, i.e., allowing selective and sequential release of predefined amounts of liquid. At a given rotational speed, the first membrane i.e., the membrane closer to disc center stretches and liquid is released through the first aperture. After liquid release the membrane reverts back to its previous state and stops the flow due to changes in liquid level in the micro-pipette (Δz). The spinning speed is increased until there is no liquid left behind the first aperture. In the same manner the liquid remaining in the micro-dispenser is released through the second aperture by further increasing the spinning speed.

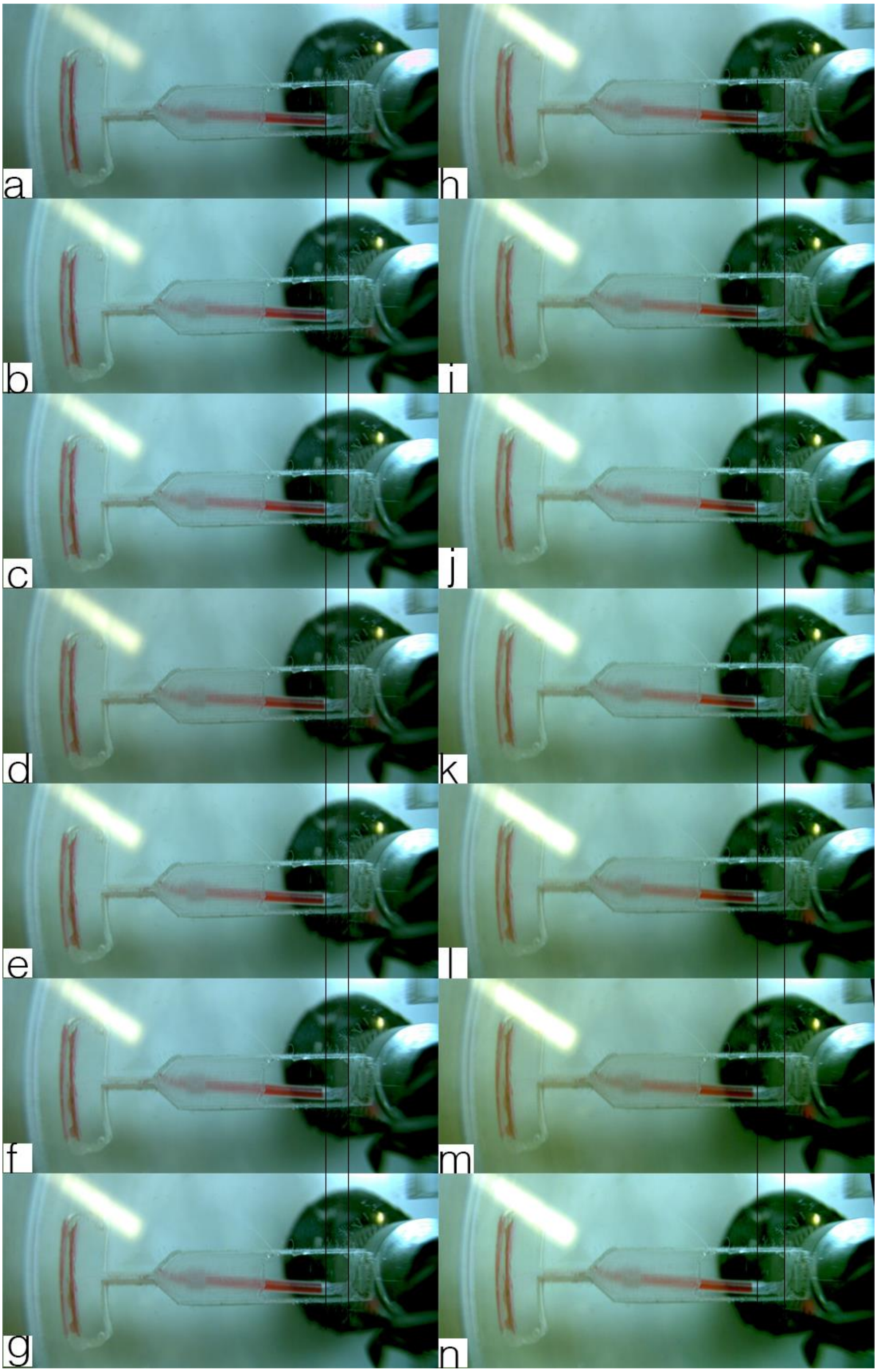

Supplementary Figure 1: Sequential and controlled release of a micro-dispenser initially containing 19µl red dyed di-water, the four accessory grid lines are to help visualization of the liquid displacement

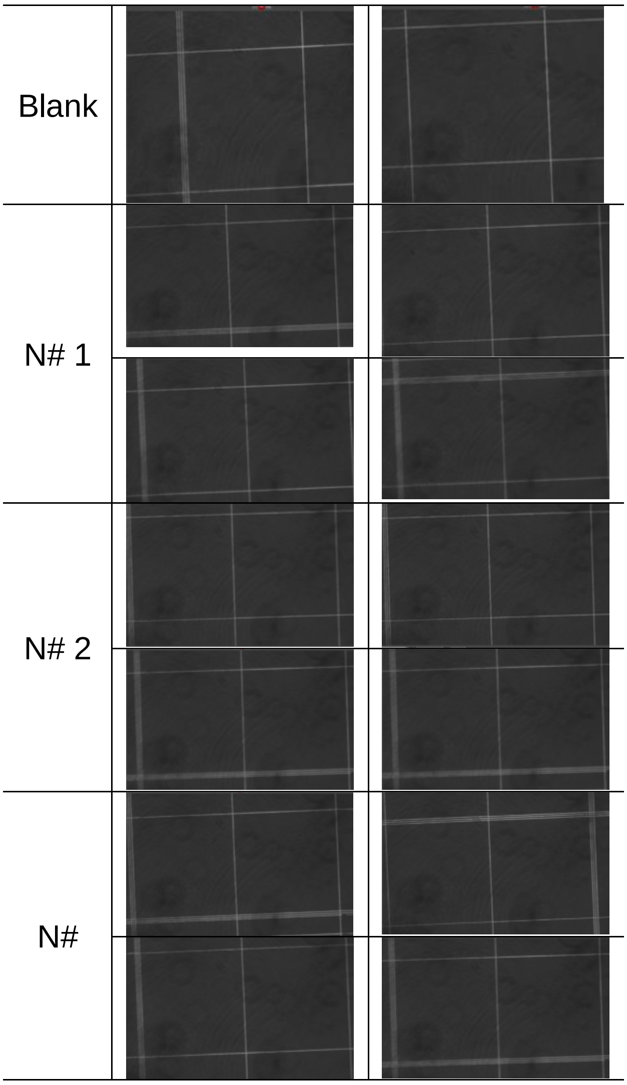

Supplementary Figure 2 Hemocytometry test, Images from plasma samples collected using micro-dispenser

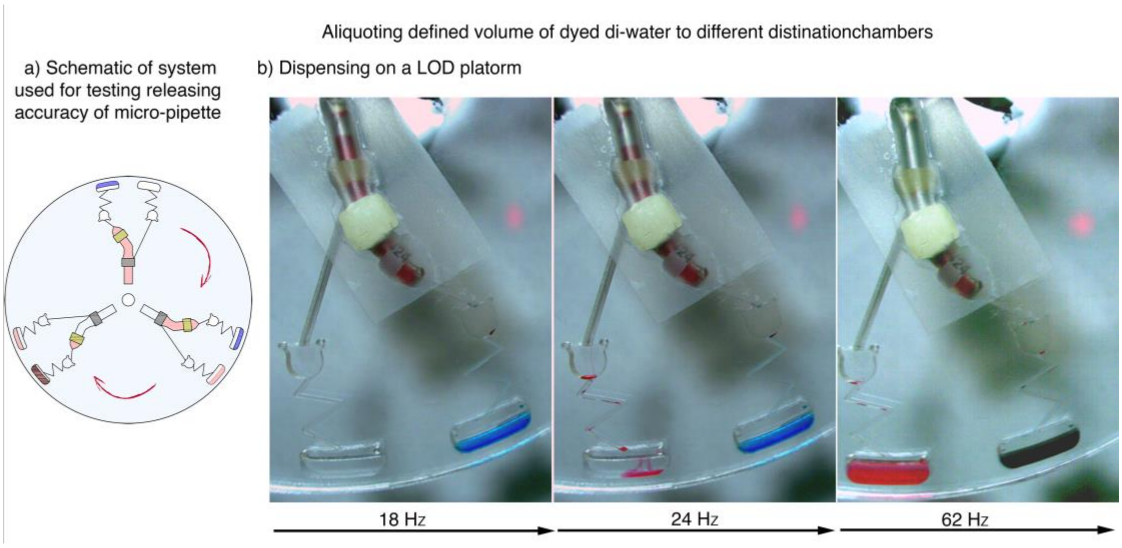

Supplementary Figure 3 a) schematic view of the platform used for aliquoting red dyed di-water in two receiving chambers (one field with di-water another with blue dye) b) experimental images of the aliquoting test.
